# Biocontrol Strains Differentially Shift the Genetic Structure of Indigenous Soil Populations of *Aspergillus flavus*

**DOI:** 10.3389/fmicb.2019.01738

**Published:** 2019-07-31

**Authors:** Mary H. Lewis, Ignazio Carbone, Jane M. Luis, Gary A. Payne, Kira L. Bowen, Austin K. Hagan, Robert Kemerait, Ron Heiniger, Peter S. Ojiambo

**Affiliations:** ^1^Department of Entomology and Plant Pathology, Center for Integrated Fungal Research, North Carolina State University, Raleigh, NC, United States; ^2^Department of Entomology and Plant Pathology, Auburn University, Auburn, AL, United States; ^3^Department of Plant Pathology, University of Georgia, Coastal Plain Experiment Station, Tifton, GA, United States; ^4^Department of Crop Science, North Carolina State University, Raleigh, NC, United States

**Keywords:** aflatoxin, *Aspergillus* section *Flavi*, biological control, lineage, mating type

## Abstract

Biocontrol using non-aflatoxigenic strains of *Aspergillus flavus* has the greatest potential to mitigate aflatoxin contamination in agricultural produce. However, factors that influence the efficacy of biocontrol agents in reducing aflatoxin accumulation under field conditions are not well-understood. Shifts in the genetic structure of indigenous soil populations of *A. flavus* following application of biocontrol products Afla-Guard and AF36 were investigated to determine how these changes can influence the efficacy of biocontrol strains in reducing aflatoxin contamination. Soil samples were collected from maize fields in Alabama, Georgia, and North Carolina in 2012 and 2013 to determine changes in the population genetic structure of *A. flavus* in the soil following application of the biocontrol strains. *A. flavus* L was the most dominant species of *Aspergillus* section *Flavi* with a frequency ranging from 61 to 100%, followed by *Aspergillus parasiticus* that had a frequency of <35%. The frequency of *A. flavus* L increased, while that of *A. parasiticus* decreased after application of biocontrol strains. A total of 112 multilocus haplotypes (MLHs) were inferred from 1,282 isolates of *A. flavus* L using multilocus sequence typing of the *trpC, mfs*, and AF17 loci. *A. flavus* individuals belonging to the Afla-Guard MLH in the IB lineage were the most dominant before and after application of biocontrol strains, while individuals of the AF36 MLH in the IC lineage were either recovered in very low frequencies or not recovered at harvest. There were no significant (*P* > 0.05) differences in the frequency of individuals with *MAT1-1* and *MAT1-2* for clone-corrected MLH data, an indication of a recombining population resulting from sexual reproduction. Population mean mutation rates were not different across temporal and spatial scales indicating that mutation alone is not a driving force in observed multilocus sequence diversity. Clustering based on principal component analysis identified two distinct evolutionary lineages (IB and IC) across all three states. Additionally, patristic distance analysis revealed phylogenetic incongruency among single locus phylogenies which suggests ongoing genetic exchange and recombination. Levels of aflatoxin accumulation were very low except in North Carolina in 2012, where aflatoxin levels were significantly (*P* < 0.05) lower in grain from treated compared to untreated plots. Phylogenetic analysis showed that Afla-Guard was more effective than AF36 in shifting the indigenous soil populations of *A. flavus* toward the non-toxigenic or low aflatoxin producing IB lineage. These results suggest that Afla-Guard, which matches the genetic and ecological structure of indigenous soil populations of *A. flavus* in Alabama, Georgia, and North Carolina, is likely to be more effective in reducing aflatoxin accumulation and will also persist longer in the soil than AF36 in the southeastern United States.

## Introduction

*Aspergillus flavus* and *Aspergillus parasiticus* are considered the most important aflatoxin-producing species within *Aspergillus* section *Flavi* (Klich, 2007). Aflatoxin production by these two *Aspergillus* species contaminates major food crops and tree nuts and thus, consumption of contaminated products poses a health hazard to humans and animals globally (Williams et al., 2004). Aflatoxins are classified as a group 1 carcinogen by the International Agency for Research on Cancer (IARC, 2002). In humans, chronic exposure to aflatoxins can result in suppression of the immune system, teratogenicity and retardation of growth in children (Richard and Payne, 2003; Paulussen et al., 2016). In maize, aflatoxins can form in kernels during crop development if the crop is stressed by heat or drought or if the crop is damaged by insects. Accumulation of aflatoxins can also occur after crop maturation when the crop is exposed to temperature and moisture conditions that are conducive to infection by *A. flavus* post-harvest and in storage (Payne, 1992). Due to the food safety concerns associated with aflatoxin contamination, more than 100 countries including the United States, have set stringent regulatory levels for quantities of aflatoxin in food and feed. The economic impact from aflatoxin contamination in the United States is primarily due to market loss and is estimated to be several hundred million dollars (Wu and Guclu, 2012).

Pre-harvest strategies such as planting resistant cultivars, good cultural practices, and biocontrol control are some strategies that are being investigated to control aflatoxin contamination (Ojiambo et al., 2018). Plant breeding efforts over the last 25 years have not provided adequate levels of resistance to aflatoxin accumulation in maize (Warburton and Williams, 2014). Environmental conditions drive aflatoxin accumulation in several crops by simultaneously affecting the population structure and virulence of *A. flavus* and the susceptibility of the host crop (Munkvold, 2003). These environmental factors continue to pose huge challenges in breeding for aflatoxin resistance due to the large genotype-by-environment interaction (Warburton and Williams, 2014; Bhatnagar-Mathur et al., 2015), an observation that has greatly limited the utility of any available resistant germplasm for the control of aflatoxin accumulation in maize. Of all the above pre-harvest strategies, biocontrol involving the application of non-aflatoxigenic strains of *A. flavus* at high densities in the field, offers the greatest potential in the mitigation of aflatoxin accumulation especially in the near-term (Dorner, 2004; Bhatnagar-Mathur et al., 2015). Non-aflatoxigenic strains are usually applied in the field using inoculated or coated cereal grains but other sprayable formulations that utilize bioplastics instead of grains, have also been developed (Abbas et al., 2017). The type of formulation used for the biocontrol product can also affect the quantity of inoculum applied on the crop (Accinelli et al., 2016). Through competitive exclusion, biocontrol strains exclude native, aflatoxigenic strains from the crop, thereby reducing aflatoxin accumulation (Dorner, 2004). Application of non-aflatoxigenic strains of *A. flavus* as biocontrol strains has reduced aflatoxin contamination in maize, cotton, and peanut by 67–95% (Cotty and Bayman, 1993; Dorner, 2008; Atehnkeng et al., 2014; Mauro et al., 2018). In the United States, Afla-Guard and AF36, are two commercial biocontrol products containing non-aflatoxigenic strains of *A. flavus* that have been approved by the U.S. Environmental Protection Agency for biocontrol of aflatoxin accumulation in peanut, maize, and cottonseed. The non-aflatoxigenic strain in Afla-Guard is NRRL 21882, which was originally isolated from a naturally infected peanut in Georgia (Dorner, 2004). The non-aflatoxigenic strain in AF36 is NRRL 18543, which was isolated from cottonseed in Arizona (Cotty, 1989). The *A. flavus* strain in Afla-Guard does not produce aflatoxins or cyclopiazonic acid (CPA) and belongs to the IB lineage, which is also composed of *A. flavus* L-strains that do not produce or are low producers of aflatoxins and strains of *A. oryzae* (Geiser et al., 2000). Unlike the Afla-Guard strain, the AF36 strain has a full aflatoxin gene cluster with one defective gene and a functional CPA cluster and belongs to the IC lineage that is composed of both aflatoxigenic and non-aflatoxigenic members (Geiser et al., 2000; Moore et al., 2009).

The logic behind the effectiveness of biocontrol using non-aflatoxigenic strains of *A. flavus* is based on the assumption that these strains are predominantly asexual, genetically stable and thus, unable to recombine with native aflatoxigenic strains (Ehrlich and Cotty, 2004; Abbas et al., 2011a). However, subsequent studies have provided unequivocal evidence for recombination within the aflatoxin gene clusters in *A. flavus* and *A. parasiticus* populations (Horn et al., 2009a,b; Moore et al., 2009) within the same field. Such a process could result in reduced or increased efficacy of the non-aflatoxigenic *A. flavus* due to the production of novel *A. flavus* phenotypes, resulting in greater diversity in the field (Fisher and Henk, 2012). The presence of high population densities of *A. flavus* during deployment of biocontrol strains can also increase opportunities for sexual recombination and re-assortment of genes that could further influence the competitiveness between strains and their capacity to produce aflatoxin (Olarte et al., 2012). This is particularly important where the biocontrol strain is genetically different from the predominant local populations of *A. flavus* in the soil.

Field populations of *A. flavus* are highly diverse (Ehrlich et al., 2015) and the genetic structure of *A. flavus* differs greatly across the United States. For example, the population in North Carolina is predominately clonal with a high frequency of the IB lineage, while that in Texas has a high frequency of the IC lineage (Horn and Dorner, 1999; Jaime-Garcia and Cotty, 2010). Afla-Guard has been reported to significantly reduce aflatoxin accumulation to a greater extent than AF36 in Mississippi (Abbas et al., 2011a,b). Similarly, Afla-Guard was found to be more effective than AF36 in reducing aflatoxin accumulation on maize in North Carolina (Meyers et al., 2015). In contrast, AF36 seems to be more effective than Afla-Guard in reducing aflatoxin accumulation in Texas (Outlaw et al., 2014). Although statistically significant differences between these two biocontrol strains in their ability to reduce aflatoxin accumulation has not been observed in all locations tested, prevailing evidence suggests that the relative effectiveness of the two biocontrol strains depends on the location where they are applied. Our working hypothesis is that the genetic composition of the indigenous soil population of *A. flavus* dictates the relative effectiveness of biocontrol strains in reducing aflatoxin contamination. This implies that understanding the genetic structure of *A. flavus* soil populations will enable the selection of biocontrol strains most similar, genetically, to the predominant indigenous multilocus haplotype (MLH) and thus, improve the efficacy of biocontrol (Ehrlich, 2014; Ehrlich et al., 2015).

Application of non-aflatoxigenic biocontrol strains that are genetically similar to local *Aspergillus* soil communities in the soil is not only considered efficacious, but maximizes the potential for sexual recombination. A non-aflatoxigenic strain that is genetically similar to native strains should increase the efficacy of biocontrol and minimize the risk of aflatoxin contamination (Bhatnagar-Mathur et al., 2015; Molo et al., 2019). The overall goal of this study was to establish the impact of the genetic structure of *A. flavus* populations in the soil on the efficacy of biocontrol of aflatoxin accumulation in maize. The specific objectives of this study were to: (i) characterize the temporal distribution of species of *Aspergillus* section *Flavi* following application of either Afla-Guard or AF36 in the field, (ii) determine the dynamics and shifts in predominant MLHs of *A. flavus* in soil treated with Afla-Guard or AF36, and (iii) inform selection of biocontrol strains and infer their effectiveness based on shifts in the frequency of indigenous MLHs of *A. flavus* in the soil. Insights in how well biocontrol strains establish in a field relative to indigenous populations of *A. flavus* can be useful in the selection of the most effective non-aflatoxigenic strains that will result in sustainable biocontrol of aflatoxin accumulation (Ehrlich et al., 2015).

## Materials and Methods

### Description of Field Sites

Field experiments were conducted during the maize growing season in 2012 and 2013 in the southeastern United States in Alabama, Georgia and North Carolina. In 2012, trials were located at the Gulf Coast Research and Extension Center in Fairhope, Alabama, and in Ben Hill County, Georgia. In 2013, trials were conducted at the Prattville Agricultural Research Unit in Prattville, Alabama and at the Coastal Plain Experiment Station in Tifton, Georgia. In North Carolina, the 2012 and 2013 field experiments were conducted at the Upper Coastal Plain Research Station in Rocky Mount. In Alabama, the maize hybrids Pioneer 31P42 and DKC 67-88 were used in 2012 and 2013, respectively, while in Georgia, the maize hybrids Pioneer 33M52 and DK 66-94 were used in 2012 and 2013, respectively. The maize hybrid DKC 64-69 was used in 2012 and 2013 in North Carolina. Standard field plots measuring 51 m wide × 69 m long with 1.5 m borders were adopted in all the three states in both years. The northern-most location of field plots in Georgia was at 31° 25′ 50″ N, −83° 32′ 10″ W, in Alabama at 32° 27′ 30″ N, −86° 34′ 36″ W, and in North Carolina at 35° 53′ 59″ N, −77° 40′ 31″ W.

### Treatments and Experimental Design

Two commercially available biocontrol products, Afla-Guard and AF36, were evaluated in this study to determine how the dynamics of dominant MLHs of *A. flavus* in the soil can influence the efficacy of biocontrol in reducing aflatoxin contamination in maize. Afla-Guard contains *A. flavus* strain NRRL 21882 as the active ingredient and is labeled for use on peanuts and maize in the United States (U.S. Environmental Protection Agency, 2013). The *A. flavus* strain in AF36 is NRRL 18543 and the product is labeled for use on maize in Arizona and Texas and on cotton in Arizona, California, and Texas (U.S. Environmental Protection Agency, 2011). Afla-Guard and AF36 were evaluated in North Carolina in 2012 and 2013 and in Alabama in 2013, while only Afla-Guard was evaluated in field plots in Alabama in 2012 and Georgia in 2012 and 2013.

Field plots were established on 21 March 2012 and 2 April 2013 in Alabama, on 10 July 2012 and 1 May 2013 in Georgia. In North Carolina, plots were planted on 3 April 2012 and 11 April 2013. Fertilization and weed control practices were used at each field site according to standard management practices for maize growers in each state. Afla-Guard and AF36 treatments were applied mechanically or manually by broadcasting the biocontrol product at recommended label rates on top of the plant canopy at the VT growth stage. In 2012, treatments were applied on 24 May, 11 May, and 16 May in Alabama, Georgia, and North Carolina, respectively. Treatment application dates in 2013 were 26 June, 8 June, and 21 June, in Alabama, Georgia, and North Carolina, respectively. Based on the number of biocontrol products, three treatments (Afla-Guard, AF36, and untreated control) were evaluated in North Carolina in both years and in Alabama in 2013. Two treatments (Afla-Guard and untreated control) were evaluated in Alabama in 2012 and Georgia in 2012 and 2013. In all states, the experiment was laid out in a randomized complete block design with three to four replications. Weather data at each experimental site during the study period were obtained from the nearest state weather station or from the national weather database at the NC State Climate Office in Raleigh, North Carolina (http://www.nc-climate.ncsu.edu/cronos).

### Soil Sampling in Experimental Fields

From each field, 20 soil samples (~100 g each) were collected using sterile plastic scoops from 20 georeferenced points at approximately equal distances along two diagonals of the field. During the study, soil samples were taken at three sampling periods: (1) prior to application of biocontrol treatments, (2) 1–2 weeks after application of biocontrol treatments, and (3) at harvest. In North Carolina, soil samples from the three sampling periods were collected on 23 May, 12 July, and 17 September 2012, respectively, while in 2013 the samples were collected on 26 June, 5 July, and 5 September 2013. In the 2012 trial in Alabama, soil samples were collected on 24 May, 18 June, and 7 September, while soil samples were collected on 2 July, 23 August, and 20 September in 2013. In Georgia, soil samples were collected on 18 May 2012 and 15 June in 2012 and no samples were collected at harvest due to flooding of the field. In the 2013, soil samples were collected from two time periods: before application of treatments on 28 May 2013 and after harvest on 21 Feb 2014. After each sample collection, soils were placed in doubled-layered brown paper bags and dried on a laboratory bench for 1–2 weeks. Soil samples collected from Alabama and Georgia were then shipped to NC State University in Raleigh and refrigerated at 4°C until further processing.

### Fungal Isolation, Identification, and Determination of Colony Forming Units

Each soil sample was first homogenized manually by shaking the contents in the sampling bag for 1 min. A sample of 33 g of soil was taken from each paper bag and added to 100 mL of 0.2% water agar and the mixture was carefully shaken for 1 min. The soil-water agar suspension was then plated on modified dichloran Rose Bengal (mdRB) medium as described by Horn and Dorner (1998). Briefly, aliquots of 200–400 μl of the soil-agar suspension were spread on the surface of mdRB medium in 100 × 15 mm diameter Petri dishes and the dishes were incubated at 37°C for 3 days. The actual volume of soil solution plated on the mdRB plates varied between samples of soil-agar suspension, so the appropriate aliquot volume was determined by experimenting with the soil to 0.2% water agar ratio (data not shown).

Total colony counts were recorded as described previously (Horn and Dorner, 1998) based on five replicate plates of each soil sample. Colonies of *Aspergillus* were identified at the species level based on conidial color along with the colony shape and colony morphology (Klich and Pitt, 1988; Cotty, 1989). Confirmation of the identity of the species of isolated colonies was determined using NCBI Standard Nucleotide BLAST search tool based on sequenced DNA fragments at the *trpC* locus (Olarte et al., 2012). Final colony-forming units (CFU) per gram of soil were corrected for soil moisture content and expressed on a dry weight soil basis. At each soil sampling period, single spores of 20 isolates of *A. flavus* were randomly picked from 20 soil dilution plates, transferred onto 60 × 15 mm Petri dishes containing mdRB medium and incubated for 5 days. This resulted in 400 isolates of *A. flavus* from each field at each sampling period in each state. A total of 6,400 isolates of *A. flavus* were obtained across the study and subjected to genetic and molecular characterization as described below. Isolates were subjected to short-term storage on mdRB medium at 4°C, while a suspension of spores in a 40% glycerol was stored at −80°C for long term storage.

### DNA Extraction and Multilocus Sequence Typing

DNA was extracted using the CTAB method (He et al., 2007) from spores harvested directly from single-spore culture colonies of 6,400 isolates of *A. flavus* grown on mdRB medium. Using PCR amplification, 80–90 *A. flavus* isolates from each sampling period in each state for both years were randomly selected for MLH diversity analysis using multilocus sequence typing (MLST). Genome-wide variation was examined using MLST based on variation at three loci; microsatellite marker AF17 on chromosome 2 (Grubisha and Cotty, 2009), major facilitator superfamily *mfs* gene on chromosome 3, and tryptophan synthase (*trpC*) gene on chromosome 4. Multilocus sequence typing was conducted for both clone corrected and uncorrected mating-type (*MAT)* data (Olarte et al., 2012). Sequences of oligonucleotide primers (*trpC, mfs*, AF17, *MAT*) and thermocycler conditions used in this study were adopted from those previously described by Carbone et al. (2007) and Olarte et al. (2012). Reactions were run 5 min at 94°C followed by 40 cycles for 30 s at 60°C for *mfs*, 58°C for *trpC, MAT1-1*, and *MAT1-2*, and 57°C for AF17, ending with 1 min at 72°C. Multiplex-PCR was used to determine the mating-type of each isolate using the *MAT1-1* and *MAT1-2* primers (Ramirez-Prado et al., 2008). All the sequencing work was performed at the NC State University Genomic Sciences Laboratory in Raleigh, North Carolina.

DNA sequences were aligned and manually adjusted using Sequencher Version 4.7 (Gene Codes Corporation, Ann Arbor, MI, USA). Alignments were exported as NEXUS files into the Mobyle SNAP Workbench (http://snap.hpc.ncsu.edu/), a web-based analysis portal deployed at NC State University (Monacell and Carbone, 2014). The SNAP Convert tool (Aylor et al., 2006) implemented in Mobyle SNAP workbench was used to convert NEXUS files into PHYLIP format. Multiple sequence alignments for each locus were combined using SNAP Combine (Aylor et al., 2006) and collapsed using SNAP Map for inference of MLHs. For maximal MLH resolution, collapsing into MLHs was performed with the option of recoding insertions/deletions (i.e., indels).

### Population Genetics, Structure, and Phylogenetic Analyses

Population summary statistics per locus were generated to infer different genetic aspects of populations of *A. flavus* isolates collected at different sampling periods in this study. These statistics included: (1) number of segregating sites (*s*), (2) average pairwise difference between sequences, π, based on Nei and Li (1979), and (3) Watterson's θ (Watterson, 1975) as implemented in ARLEQUIN v3.5 (Excoffier and Lischer, 2010). Tajima's *D* (Tajima, 1989) and Fu's F_S_ (Fu and Li, 1993) were used as tests of neutrality and population size constancy. Input files for calculating these population summary statistics were generated using SNAP Map excluding indels and assuming an infinite-sites model of DNA sequence evolution. The phylogenetic relationship of 1,282 isolates was examined for each locus separately and for the combined multi-locus dataset using maximum likelihood analysis implemented in RAxML (Stamatakis et al., 2008) through the CIPRES RESTful application programming interface (API) (Miller et al., 2015) implemented in the SNAP Portal. Confidence limits on branches in phylogenies were based on 1,000 rapid bootstrap replicates and monophyletic groups were identified as branches having at least 70% bootstrap support. Phylogenetic trees were visualized using the Tree-Based Alignment Selector (T-BAS) v2 toolkit (Carbone et al., 2017, 2019).

Multilocus sequence variation was further subjected to analysis of molecular variance (AMOVA) to test the null hypothesis that populations were not genetically differentiated over the multiple hierarchical spatial scales or among distinct sampling periods. AMOVA was used to estimate the genetic variance components at different hierarchical levels of population structure (Excoffier and Lischer, 2010) and the pairwise fixation index (*F*_*ST*_) was calculated to quantify genetic differentiation within and among *A. flavus* populations. Significance of *F*_*ST*_ analyses was determined using 1,000 permutations in ARLEQUIN v3.5. Structure was also examined using principal component analysis (PCA) and the methods described in Patterson et al. (2006) implemented in the Mobyle SNAP Workbench. Principal components were normalized to sum to 1, and the number of significant axes of variation (i.e., principal components or eigenvectors) was determined using the Tracy–Widom statistic (Tracy and Widom, 1994). The optimal number of clusters for *k*-means was determined using the cluster center initialization algorithm that centers on randomly chosen observed points (Khan and Ahmad, 2004). Clusters were evaluated using the Calinski–Harabasz index (Calinski and Harabasz, 1974), which identifies the best cluster based on the average between and within cluster sum of squares. Significant principal components and clusters were displayed graphically using the SCATTERPLOT3D package in R (Ligges and Mächler, 2003). We used Fisher's exact test implemented in the Mobyle SNAP Workbench to determine if there were non-random associations between cluster and state, year, or sampling period.

Phylogenetic incongruence across *trpC, mfs*, and AF17 was examined using patristic distances displayed as a heat map in outer rings (one per locus) in T-BAS v2.1 (Carbone et al., 2019). For each separate locus phylogeny, a matrix of patristic distances, normalized to a maximum value of 1, was generated for all pairs of sequences representing individual isolates. The distances from different loci were compared to identify incongruences in tree topologies that suggest genetic exchange and recombination. Alternatively, congruent distances across topologies suggest clonal transmission and adaptation. Patristic distances from Afla-Guard or AF36 were displayed in T-BAS to compare patterns of phylogenetic incongruence between *trpC, mfs*, and AF17.

### Mating-Type Distribution of *A. flavus* Isolates

Clone correction was performed using MLST to eliminate accidental sampling of the same individual multiple times (Moore et al., 2013). In this study, the null hypothesis was that there is no significant difference between the frequencies of *MAT1-1* and *MAT1-2* individuals at each sampling time period in each state and experimental year, which would indicate frequency-dependent selection consistent with sexual reproduction (Linde et al., 2003). This hypothesis was tested using a two-tailed binomial test on clone corrected and clone uncorrected data sets for variation at three MLST loci, *trpC, mfs*, and AF17, using the binomial option in PROC FREQ in SAS (version 9.4; SAS Institute, Cary, NC). A significant difference in the frequency of the two mating-types before and after clone correction would indicate a primarily asexual population. In contrast, a significant difference in the frequency of the two mating-types before clone correction and a lack of no significant difference after clone correction, or a lack of significant difference for either the uncorrected or corrected population, would suggest that the fungal population is predominantly undergoing sexual reproduction (Leslie and Klein, 1996; Linde et al., 2003).

### Quantification of Aflatoxin in Harvested Grain

At each location, a subsample of about 2.5 kg of harvested grain dried to 15–17% moisture content was randomly selected for enumeration of aflatoxin contamination. Due to logistic and environmental constraints, harvesting was not conducted in Georgia in 2012 and thus, no data on aflatoxin contamination in the field was obtained. Aflatoxin was quantified in harvested grain in Georgia and North Carolina using the VICAM column system as described by Truckness et al. (1991) and the detection limit for the VICAM method is 5 ppb. The Veratox aflatoxin kit (Neogen Corporation, Lansing, MI), which has a detection limit of 2 ppb, was used according to kit instructions to quantify aflatoxin in harvested grain in Alabama as described by (Bowen et al., 2014).

### Analysis of Soil Population Densities and Aflatoxin Contamination in Grain

Based on preliminary data analyses, data for soil population densities recorded as colony forming units per g of soil (CFU/g) and aflatoxin concentration (ppb) in harvested grain were analyzed separately for each state and year. Means CFU were calculated at each sampling period and the range was used to depict the soil population densities of various members of *Aspergillus* section *Flavi* at different sampling periods within each state. Means of aflatoxin concentration from each treatment plot were subjected to analysis of variance using the PROC GLM of SAS. Fisher's LSD test (α = 0.05) was used to separate means of aflatoxin concentration between biocontrol treatments evaluated in each state.

## Results

### Weather Conditions

Weather factors recorded during the study period varied between years and experimental sites. In both years, temperatures during the growing season increased from April to July at all experimental sites (Table 1). In 2012, the highest temperatures were recorded at Rocky Mount in North Carolina that had a maximum temperature of 34°C with a mean temperature of 32°C between April and July. In 2013, the highest temperatures were recorded at Prattville, Alabama with a maximum temperature of 31°C and a mean temperature of 29°C between April and July. The lowest maximum temperatures in 2012 were recorded at Ben Hill in Georgia with a mean temperature of 29°C from April to July, while the corresponding lowest temperatures in 2013 were recorded at Rocky Mount in North Carolina and Tifton in Georgia with a mean of 28°C (Table 1).

**Table 1:** Summary of weather variables recorded at experimental sites in a study conducted to assess the impact biocontrol strains on genetic structure of *Aspergillus flavus* in the field.

	**2012**			**2013**		
**Variable/month**	**North Carolina**	**Alabama**	**Georgia**	**North Carolina**	**Alabama**	**Georgia**
**MEAN MAX/MIN TEMPERATURE (°C)**						
April–May	28/12	28/16	26/14	24/12	26/13	26/14
June–July	35/19	31/22	32/20	31/20	31/21	30/22
Mean^a^	32/16	30/19	29/17	28/16	29/17	28/18
**RAINFALL (mm)**						
April–May	259	193	88	141	145	181
June–July	236	399	212	378	465	384
Total^b^	495	592	300	519	610	565

a *Mean temperature recorded from April to end of July*.

b *Total amount of rain recorded from April to end of July*.

Rainfall amounts during the season were lower in 2012 than in 2013, with the Ben Hill in Georgia being the driest site in 2012 with 300 mm from April to July, while Rocky Mount in North Carolina was the driest site in 2013 with 519 mm. The wettest sites in 2012 and 2013 were Fairhope and Prattville both in Alabama with 592 and 610 mm, respectively, being recorded from April to July (Table 1).

### Soil Population Densities of *Aspergillus* Section *Flavi*

Soil densities of *Aspergillus* section *Flavi* in the soil increased over time following the application of biocontrol treatments in both years across the three states except in Georgia in 2012 (Table 2). Densities were lowest prior to the application of treatments and highest at harvest in Alabama, Georgia (in 2013), and North Carolina, with the densities at the pre-application sampling period being intermediate. For example, the mean soil population densities at pre-application, post-application, and harvest in North Carolina in 2012 were 38, 237, and 986 CFU/g, respectively, while the corresponding populations in 2013 were 157, 240, and 250 CFU/g, respectively. In 2012, the lowest minimum population density was 3 CFU/g in soil samples from Alabama prior to the application of biocontrol treatments, while the highest maximum population density of 3,019 CFU/g was observed in Alabama at harvest. In 2013, the lowest minimum population density was 1 CFU/g in soils from Georgia prior to treatment application, while the highest maximum soil density of 1,406 CFU/g was observed at harvest in Alabama (Table 2).

**Table 2:** Population densities of *Aspergillus* section *Flavi* in soil from fields in the southeastern United States treated with Afla-Guard and AF36 biocontrol strains.

		Colony forming units (CFU) at sampling period^a^			ΔCFU^b^	
**Year**	**State**	**Pre-application**	**Post-application**	**Harvest**	**Post-application**	**Harvest**
2012	Alabama	33 (3–189)	151 (7–679)	516 (33–3,019)	4.6	15.6
	Georgia	413 (4–1,906)	220 (9–888)	–^c^	−0.5	–^c^
	North Carolina	38 (11–113)	237 (6–1786)	986 (21–1,005)	6.2	25.9
2013	Alabama	106 (16–212)	111 (42–227)	376 (48–1,406)	1.1	3.5
	Georgia	20 (1–103)	–^c^	173 (16–432)	–^c^	8.6
	North Carolina	157 (6–509)	240 (3–1,009)	250 (3–926)	1.5	1.6

a *Soil densities (i.e., CFU) are means per gram of soil based on 20 samples collected from each field in a state. Numbers in parenthesis represent the range (minimum to maximum) of CFU. AF36 and Afla-Guard were evaluated in both years in North Carolina. In Alabama, Afla-Guard was evaluated in both years, while AF36 was evaluated only in 2013. In Georgia, only Afla-Guard was evaluated in both years*.

b *ΔCFU refers to change (– or +) in CFU relative to CFU prior to application of biocontrol strains. ΔCFU = (x/y), where x = CFU at post-application or harvest, and y = CFU at pre-application of biocontrol strains*.

c *Soil samples were not collected at this time period and no data is available*.

Application of biocontrol treatments also impacted the densities of *A. flavus* in the soil. This impact was more pronounced in 2012 than in 2013 and at harvest than at post-inoculation (Table 2). In addition, this impact was also observed in Alabama and North Carolina in 2012 and Georgia in 2013. For example, the change in soil populations following the application of biocontrol (i.e., ΔCFU) in North Carolina at post-application was about 4-fold higher in 2012 compared to 2013. This pattern was observed across all three states for both years except 2012 in Georgia, where ΔCFU decreased at post-application. In North Carolina, ΔCFU at harvest was about 4- and 1.1-fold higher than at post-application in 2012 and 2013, respectively. This same pattern was also observed in Alabama but with much higher values in both years (Table 2).

### Frequency of Species Within *Aspergillus* Section *Flavi*

Within *Aspergillus* section *Flavi, A. flavus, A. parasiticus, A. caelatus, A. nomius*, and *A. tamarii* were recovered from soil collected from the study sites across three states. However, the incidence of individual species varied between states, with the diversity within section *Flavi* being higher in Alabama compared to Georgia and North Carolina (Table 3). In addition, the incidence of members within *Aspergillus* section *Flavi* in each state was fairly consistent in both years of the study. Across all the states, *A. flavus* was the dominant species with a frequency of 61–100%. In addition, all *A. flavus* isolates sampled in Alabama, Georgia and North Carolina belonged to the L-strain morphotype. The highest proportion of *A. flavus* across sampling periods was observed in Georgia (97.9–100%), followed by North Carolina (84.9–96.8%) and Alabama (61.0–98.0%; Table 3).

**Table 3:** Frequency of members within *Aspergillus* section *Flavi* isolated from soil in fields in southeastern United States treated with Afla-Guard and AF36 biocontrol strains.

				**Incidence (%)**				
State^a^	Year	Soil sampling period	Number evaluated	*A. flavus*	*A. parasiticus*	*A. caelatus*	*A. nomius*	*A. tamarii*
Alabama	2012	Pre-application	94	95.7	4.3	0.0	0.0	0.0
		Post-application	154	61.0	35.1	3.2	0.7	0.0
		Harvest	106	82.1	13.2	4.7	0.0	0.0
	2013	Pre-application	105	82.9	16.2	0.9	0.0	0.0
		Post-application	97	90.7	7.2	0.0	0.0	2.1
		Harvest	100	98.0	2.0	0.0	0.0	0.0
Georgia	2012	Pre-application	96	97.9	2.1	0.0	0.0	0.0
		Post-application	94	97.9	2.1	0.0	0.0	0.0
		Harvest	–^b^	–^b^	–^b^	0.0	0.0	0.0
	2013	Pre-application	93	97.8	2.2	0.0	0.0	0.0
		Post-application	–^b^	–^b^	–^b^	0.0	0.0	0.0
		Harvest	94	100.0	0.0	0.0	0.0	0.0
North Carolina	2012	Pre-application	106	84.9	15.1	0.0	0.0	0.0
		Post-application	94	96.8	3.2	0.0	0.0	0.0
		Harvest	94	96.8	3.2	0.0	0.0	0.0
	2013	Pre-application	105	93.3	6.7	0.0	0.0	0.0
		Post-application	94	95.7	4.3	0.0	0.0	0.0
		Harvest	97	94.8	5.2	0.0	0.0	0.0

a *Afla-Guard and AF36 were evaluated in both years in North Carolina. In Alabama, Afla-Guard was evaluated in 2012 and 2013, while AF36 was evaluated only in 2013. In Georgia, only Afla-Guard was evaluated in both years*.

b *Soil samples were not collected at this time period and no data is available*.

*Aspergillus parasiticus* was the second most abundant species observed across all states. As with *A. flavus, A. parasiticus* was found at all sampling periods in every state, except in Georgia in 2013 (Table 3). In contrast to *A. flavus*, the maximum incidence of *A. parasiticus* was highest in Alabama (35.1%) and lowest in Georgia (2.1%), with incidence in North Carolina (15.1%) being intermediate. The incidence of *A. parasiticus* was always highest prior to application of the biocontrol but decreased after the application of the biocontrol treatments with the lowest levels being observed at harvest. The only exception to this trend was in Alabama in 2012, where the incidence of *A. parasiticus* was lower at pre-application (4.3%) than at post-application (35.1%) of the biocontrol treatments. *A. caelatus, A. nomius*, and *A. tamarii* were the other species within *Aspergillus* section *Flavi* that were isolated in this study. *A. caelatus, A. nomius*, and *A. tamarii* were isolated in soils collected only from Alabama. The incidences of these three species ranged from 0 to 4.7% and were considerably lower than those observed for either *A. flavus* or *A. parasiticus*. The incidence of *A. nomius* was about 1%, while that of *A. caelatus* was about 5% of the total population across the three sampling periods. *A. tamari* was detected only in 2013 in Alabama with an incidence of 2.1%. None of these three species were isolated in soil collected at harvest (Table 3).

### Genetic Diversity in Response to Application of Biocontrol Strains

To assess shifts in the genetic structure of populations of *A. flavus* following treatment application, MLST was used to determine the number of MLHs at each soil sampling period. The number of unique MLHs varied between sampling period, states and growing seasons (Table S1). In general, the number of MLH was greater before and after the application of treatments, but lower at harvest (Table 4). A total of 112 unique MLHs were inferred in this study based on 1,282 isolates of *A. flavus* that were characterized. The highest number of unique MLHs was observed in Alabama with 73, while the number of MLHs in Georgia and North Carolina were much lower with 30 and 38, respectively (Table 4). The number of MLHs at different sampling periods in Alabama ranged from 16 in 2012 at harvest to 37 at pre-application in 2013. In North Carolina, number of MLHs ranged from 17 in 2013 at harvest to 29 in 2012 post-application of biocontrol treatments. Generally, the number of MLHs was higher in Georgia than either Alabama or North Carolina, with numbers ranging from 3 to 23 in the 2013 growing season (Table 4). Only 22 of the 112 unique MLHs were common in all three states, while MLHs unique to a specific state were highest in Alabama with 40 MLHs and considerably lower in Georgia and North Carolina that had only 7 and 16 MLHs, respectively. Sequences used for MLST (AF17, *mfs*, and *trpC*) were submitted to GenBank under accession numbers 2232583, 2233208, and 2233307.

**Table 4:** Number of unique multilocus haplotypes (MLHs) inferred from populations of *Aspergillus flavus* in soil from maize fields in southeastern United States treated with Afla-Guard and AF36 biocontrol strains in 2012 and 2013.

	Alabama^a^		Georgia^a^		North Carolina^a^	
Sampling period	2012	2013	2012	2013	2012	2013
Pre-application	16	37	21	23	23	18
Post-application	22	34	19	–^b^	29	17
Harvest	16	36	–^b^	3	19	17
Total^c^	37	73	30	25	38	32

a *AF36 and Afla-Guard were evaluated in both years in North Carolina. In Alabama, Afla-Guard was evaluated in both years, while AF36 was evaluated only in 2013. In Georgia, only Afla-Guard was evaluated in both years*.

b *Soil samples were not collected at post-application or harvest in 2013 and 2012, respectively, and no data is available*.

c *Totals are the number of unique MLHs in each year in each state. The number of unique MLHs were examined within each sampling period of each year at each location*.

**Figure 1:**
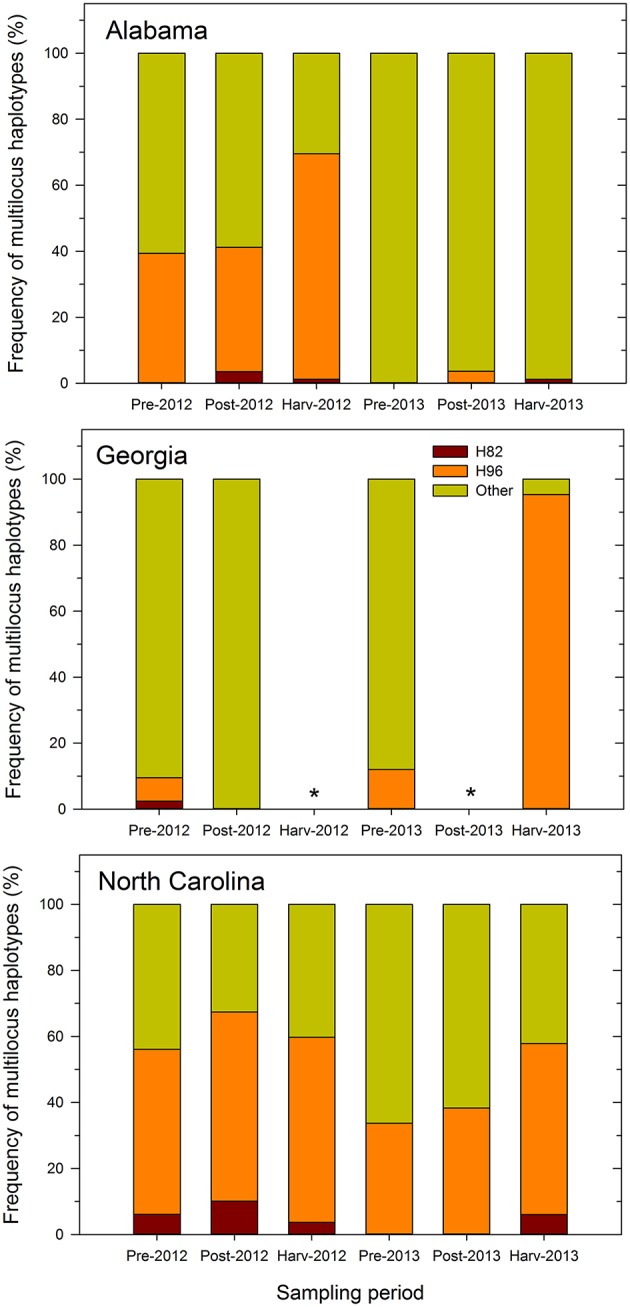
Frequency of multilocus haplotypes (MLHs) recovered (as a proportion of the total number of MLHs observed) at each sampling period from maize fields in Alabama, Georgia, and North Carolina in 2012 and 2013 using combined MLST loci (*trpC*, AF17, and *mfs*) sequence data. Pre- and post-denotes sampling time before and after application of Afla-Guard and AF36. MLHs are designated as belonging to either the Afla-Guard MLH (H96), AF36 MLH (H82), or neither of these two MHLs (Other). The asterisk (^*^) indicates that soil samples were not collected at harvest in 2012 and at post-application of the biocontrol in 2013 in Georgia and there is no corresponding MLH frequency data.

The proportion of inferred individuals that was similar to the MLH of Afla-Guard strain (H96) was higher than that of individuals similar to the MLH of AF36 strain (H82) (Figure 1). Further, the recovery of individuals belonging to the two MLHs varied by state and sampling period. For example, the proportion of individuals at different sampling periods that belonged to either H82 or H96 was less consistent across growing seasons in either Alabama or Georgia in 2012 and 2013. However, the proportions of individuals belonging to either H82 or H96 MLH prior to application of biocontrol treatments and at harvest were consistent in 2012 and 2013 in North Carolina. For example, 50 and 56% of isolates recovered in North Carolina prior to biocontrol application and at harvest, respectively, belonged to H96 in 2012. Similar levels were observed in 2013 where 34 and 52% of the isolates recovered prior to biocontrol treatment and at harvest were of the H96 MLH (Figure 1).

In Alabama, the proportions of individuals that matched either H82 or H96 varied between growing seasons. In 2012, individuals matching H96 increased over the sampling periods and ranged from 39% prior to application of the biocontrol treatments to 68% at harvest (Figure 1). In contrast, individuals belonging to H82 were fewer in 2012 and ranged between 0 and 1%. In 2013, very few individuals (1–4%) belonged to either H82 or H96. The proportion of individuals in Georgia belonging to either H82 or H96 was very low in 2012 compared to 2013. In 2012, only 2% of the recovered individuals matched the H82 and 7% of the recovered individuals belonged to H96. In 2013, no individuals recovered in Georgia belonged to the H82 haplotype, while 12 and 95% of the individuals before application of treatments and at harvest, respectively, were of the H96 MLH (Figure 1).

Recovery of *A. flavus* individuals belonging to either H82 or H96 was more consistent over the two growing seasons in North Carolina compared to either Alabama or Georgia (Figure 1). Individuals belonging to H82 and H96 were recovered in both years and at all sampling periods in North Carolina, except during the 2013 pre- and post-application periods. In 2012, most individuals recovered from the field in North Carolina belonged to H96 and they ranged from 50% at the pre-application period to 57% at the post-application period with 56% at harvest). The corresponding number of individuals belonging to H82 ranged from 6% at the harvest period to 10% at the post-application period. A similar pattern for the recovery of individuals similar to H96 in North Carolina was observed in 2013, with numbers ranging from 34% at the pre-application to 38% post-application and 52% at harvest. Individuals belonging to H82 that were recovered only at harvest in 2013 in North Carolina, accounted for only 6% of the total number of MLHs. Across the entire study, the proportion of the recovered individuals with the H96 MLH ranged from 4 to 95%, while that of individuals with H82 MLH ranged from 1 to 10% after application of treatments (Figure 1).

### Frequency and Distribution of Mating Type Genes Among Haplotypes

Based on MLH corrected data, all populations of *A. flavus* in Alabama, Georgia, and North Carolina in 2012 (Table 5) and 2013 (Table 6) did not significantly (*P* > 0.05) deviate from the 1:1 mating-type ratio except for pre-application populations in Alabama in 2012 (*P* = 0.0025) and 2013 (*P* = 0.0031). The pre-application population in Alabama in 2012 was skewed toward *MAT1-1*, while the pre-application population in 2013 was skewed toward *MAT1-2*.

**Table 5:** Frequency and distribution of mating-type (*MAT*) genes among isolates of *Aspergillus flavus* in soil from maize fields in southeastern United States treated with Afla-Guard and AF36 biocontrol strains in 2012.

			Mating-type frequency^c^		
State	Sampling period^a^	Genetic scale^b^	*MAT1-1*	*MAT1-2*	*P*-value^d^
Alabama	Pre-application	Corrected	80.8 (21)	19.2 (5)	0.0025
		Uncorrected	36.4 (32)	63.6 (56)	0.0138
	Post-application	Corrected	52.8 (19)	47.2 (17)	0.8679
		Uncorrected	38.8 (33)	61.2 (52)	0.0503
	Harvest	Corrected	59.1 (13)	40.9 (9)	0.5235
		Uncorrected	24.4 (20)	75.6 (62)	0.0001
Georgia	Pre-application	Corrected	55.2 (16)	44.8 (13)	0.7111
		Uncorrected	75.0 (63)	25.0 (21)	0.0001
	Post-application	Corrected	62.1 (18)	37.9 (11)	0.2649
		Uncorrected	69.8 (60)	30.2 (26)	0.0001
	Harvest	Corrected	–^c^	–^c^	–
		Uncorrected	–^c^	–^c^	–
North Carolina	Pre-application	Corrected	41.9 (13)	58.1 (18)	0.4731
		Uncorrected	25.6 (21)	74.4 (61)	0.0001
	Post-application	Corrected	34.6 (9)	65.4 (17)	0.1686
		Uncorrected	14.6 (13)	85.4 (76)	0.0001
	Harvest	Corrected	39.3 (11)	60.7 (17)	0.3449
		Uncorrected	23.2 (19)	76.8 (63)	0.0001

a *Denotes when soil samples were collected from the field in relation to the application of the biocontrol agents. Afla-Guard and AF36 were evaluated in both years in North Carolina. In Alabama, Afla-Guard was evaluated in both years, while AF36 was evaluated only in 2013. In Georgia, only Afla-Guard was evaluated in both years*.

b *Mating-type designation based on either uncorrected or clone corrected multilocus haplotype data*.

c *Numbers presented in parentheses refer to number of isolates examined. Soil samples were not collected at harvest in Georgia*.

d *Probability from a two-tailed exact binomial test performed under the null hypothesis of no significant difference in the frequency of isolates with MAT1-1 and MAT1-2 genes*.

**Table 6:** Frequency and distribution of mating-type (*MAT*) genes among isolates of *Aspergillus flavus* in soil from fields in the southeastern United States treated with Afla-Guard and AF36 biocontrol strains in 2013.

			Mating-type frequency^c^		
State	Sampling period^a^	Genetic scale^b^	*MAT1-1*	*MAT1-2*	*P*-value^d^
Alabama	Pre-application	Corrected	19.4 (13)	80.6 (54)	0.0031
		Uncorrected	22.5 (18)	77.5 (62)	0.0001
	Post-application	Corrected	53.1 (17)	46.9 (15)	0.8601
		Uncorrected	27.7 (23)	72.3 (60)	0.0001
	Harvest	Corrected	38.6 (17)	61.4 (27)	0.1742
		Uncorrected	25.9 (21)	74.1 (60)	0.0001
Georgia	Pre-application	Corrected	40.5 (15)	59.5 (22)	0.3240
		Uncorrected	53.0 (44)	47.0 (39)	0.6609
	Post-application	Corrected	–^c^	–^c^	–
		Uncorrected	–^c^	–^c^	–
	Harvest	Corrected	25.0 (1)	5.0 (3)	0.6250
		Uncorrected	1.2 (1)	98.8 (85)	0.0001
North Carolina	Pre-application	Corrected	56.7 (17)	43.3 (13)	0.5847
		Uncorrected	50.0 (40)	50.0 (40)	1.0001
	Post-application	Corrected	65.7 (23)	34.3 (12)	0.0895
		Uncorrected	47.6 (39)	52.4 (43)	0.7407
	Harvest	Corrected	56.0 (14)	44.0 (11)	0.6900
		Uncorrected	32.5 (26)	67.5 (54)	0.0023

a *Denotes when soil samples were collected from the field in relation to the application of the biocontrol agents. Afla-Guard and AF36 were evaluated in both years in North Carolina. In Alabama, Afla-Guard was evaluated in both years, while AF36 was evaluated only in 2013. In Georgia, only Afla-Guard was evaluated in both years*.

b *Mating-type designation is based on either uncorrected or clone corrected multilocus haplotype data*.

c *Numbers presented in parentheses refer to number of isolates examined. Soil samples were not collected at post-application of the biocontrol agent harvest in Georgia*.

d *Probability from a two-tailed exact binomial test performed under the null hypothesis of no significant difference in the frequency of isolates with MAT1-1 and MAT1-2 genes*.

Unlike with the MLH corrected data, *A. flavus* populations in Alabama significantly (*P* < 0.05) deviated from a 1:1 mating-type ratio except at the post-application population (*P* = 0.0503) when uncorrected data were analyzed using the exact binomial test (Table 5). Similar results with MLH uncorrected data were also observed for populations in Georgia and North Carolina, where all populations significantly (*P* < 0.05) deviated from a 1:1 mating-type ratio except the pre-application population (*P* = 0.6609) in 2013 in Georgia and the 2013 pre-application (*P* = 1.0000) and post-application (*P* = 0.7407) populations in North Carolina (Table 6).

### Population Genetics, Structure, and Phylogenetic Analyses

Nucleotide diversity (π) was low across the three MLST loci and estimates were similar within sampling periods in each state and ranged from 0.0002 at harvest in North Carolina to 0.0116 in Alabama prior to application of biocontrol treatments (Table 7). Tajima's *D* and Fu's *F*_*S*_ used to test the hypothesis of neutral mutation did not show significant (*P* > 0.05) deviations from neutrality except for a single population at harvest in North Carolina that showed significant (*P* < 0.05) deviation from neutrality based on the *mfs* locus (Table 7). This significant value indicates the presence of divergent alleles and balancing selection on aflatoxigenicity and non-aflatoxigenicity in the aflatoxin cluster.

**Table 7:** Neutrality based on Fu (*F*_*S*_) and Tajima (*D*) tests and nucleotide diversity estimates (π) for the three multilocus sequence typing loci for populations of *Aspergillus flavus* collected from fields in the southeastern United States treated with Afla-Guard and AF36 biocontrol strains.

**State**	**Sampling period**	*trpC*^a^			*mfs*^a^^,^^b^			AF17^a^		
		*F_S_*	*D*	π	*F_S_*	*D*	π	*F_S_*	*D*	π
Alabama	Pre-application	−1.0786	−0.7180	0.0011	−1.6917	−0.7877	0.0058	−0.4803	−0.5979	0.0116
	Post-application	−0.9824	−0.6453	0.0011	−2.0735	−0.9052	0.0048	0.9633	0.9390	0.0114
	Harvest	−1.3830	−0.7248	0.0017	−3.6820	−0.8408	0.0054	2.3024	1.0782	0.0099
	2012	−1.1333	−0.7239	0.0003	−3.7406	−0.9739	0.0042	0.2268	0.5271	0.0092
	2013	−3.0801	−1.0600	0.0020	−3.3376	−0.7285	0.0055	0.5519	−0.7717	0.0085
Georgia	Pre-application	−1.8387	−0.8155	0.0017	−1.6175	−0.3810	0.0042	−0.0881	−0.3469	0.0050
	Post-application	−1.6738	−0.8295	0.0011	−2.5014	−0.7743	0.0041	−0.3985	−0.1441	0.0044
	Harvest	−1.9450	−0.9182	0.0010	−4.0554	−1.1048	0.0036	0.1461	0.0804	0.0038
	2012	−1.2246	−0.6330	0.0013	−2.3542	−0.9817	0.0033	−0.4891	−0.4299	0.0052
	2013	−1.1754	−0.6162	0.0013	−2.6525	−0.6877	0.0046	0.1260	0.0405	0.0034
North Carolina	Pre-application	1.4768	0.6758	0.0046	−0.4811	−0.0224	0.0042	2.8917	1.7240	0.0100
	Post-application	1.5741	0.9971	0.0057	0.1346	−1.0536	0.0017	1.4757	1.3664	0.0063
	Harvest	−0.9691	−0.7874	0.0002	−1.1847	−1.5218^*^	0.0005	−1.1684	−1.2295	0.0012
	2012	2.5226	1.4506	0.0060	−2.0405	−1.0387	0.0023	1.8450	0.9399	0.0079
	2013	−2.7494	−1.3204	0.0011	−0.7275	−0.2841	0.0033	0.0532	−0.2678	0.0046

a *F_S_ measures departure from neutrality based on Fu (1997), where negative values are evidence for an excess number of alleles and suggest recent population growth, while positive values are evidence for a deficiency of alleles from a recent bottleneck; D measures departure from neutrality based on Tajima (1989), where negative values suggest rapid population growth, while positive values indicate population contraction; Nucleotide diversity (π) is based on (Nei and Li, 1979)*.

b The asterisk (^*^) denotes values with significant (P < 0.05) deviation from neutrality based on either the F_S_ or D test.

The population-scaled mean mutation rate, θ, averaged across all loci was similar in magnitude within and between state (Alabama, Georgia, North Carolina), sampling period (pre-application, post-application, harvest) and year (2012, 2013). At the state level, θ was slightly higher in in Alabama (θ = 3.747) and lower in Georgia (θ = 2.343) with values for North Carolina being intermediate (θ = 2.653). Similarly, θ differed between seasons and was 36% higher in 2013 (θ = 3.681) than in 2012 (θ = 2.710). However, no differences in θ were observed between sampling periods, where the mean θ was about 3.166. The similarity in estimates of π and θ indicates a lack of significant underlying differences in mutation rates and population genetic structure.

**Figure 2:**
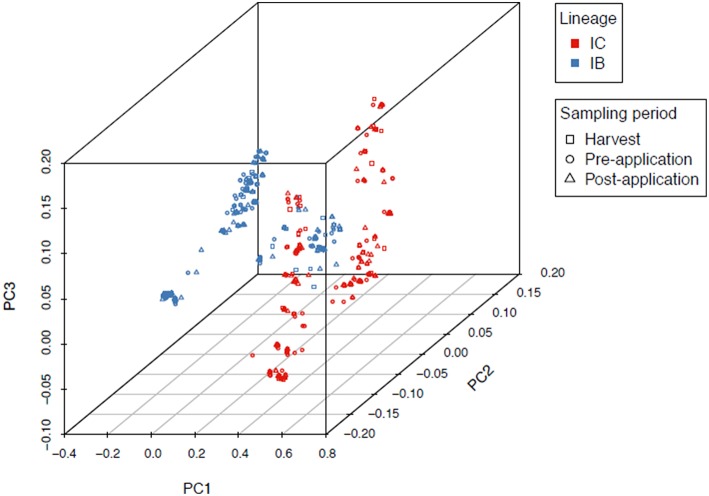
Principal component analysis of 1,282 *A. flavus* isolates showing two distinct clusters identified as lineages IB and IC based on MLST loci (*trpC*, AF17, and *mfs*). Admixture between IB and IC is indicated as a mix of red and blue lineage colors in the middle of the first principal component axis (PC1). There was a significant (*P* < 0.00001) association between lineage and sampling period.

An overall *F*_*ST*_ of 0.0089 (*P* < 0.0001) revealed very little genetic structure among sampling locations in North Carolina, Alabama, and Georgia. PCA and Tracy-Widom of MLST data identified 16 significant axes of variation. The optimal number of clusters for *k*-means ranged from 2 to 8 and the Calinski–Harabasz index found *k* = 2 as the best cluster count (Figure 2). The clusters were identified as lineages IB and IC based on sequence similarity of MLHs with previous studies (Moore et al., 2009, 2017; Olarte et al., 2012). Both the Afla-Guard (H96) and AF36 biocontrol (H82) strains were clustered in lineage IB. A two-sided Fisher's exact test showed no significant association of lineage with state (*P* = 0.07685) and year (*P* = 1.0000), but there was a significant association between lineage and sampling period (*P* < 0.00001).

**Figure 3:**
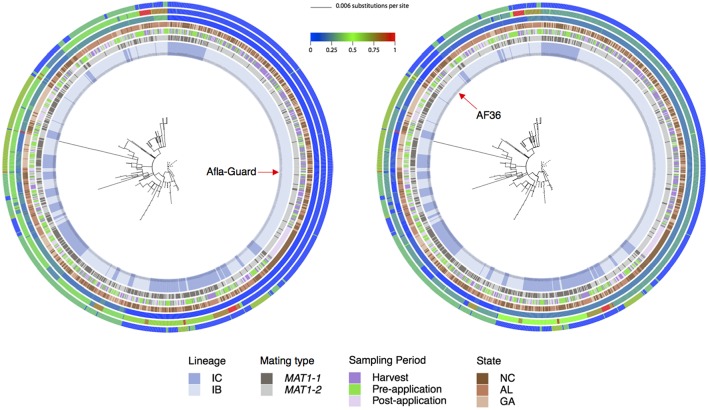
Phylogenetic relationships showing patristic distances of 1,282 *A. flavus* isolates to the Afla-Guard strain (radial tree on left) or the AF36 strain (radial tree on right). In the center of each radial ring is the best maximum likelihood tree for the combined MLST loci (*trpC*, AF17, and *mfs*) with branches drawn to scale (scale bar is shown at the top). The four innermost rings represent *A. flavus* lineage as inferred from principal component analysis, mating type, sampling period, and state, respectively. The three outermost rings represent patristic distances for AF17, *mfs*, and *trpC*, respectively. The distance of each isolate from Afla-Guard or AF36 as a reference is shown using a heat map, where a value of 0 (blue) indicates high genetic similarity of the strain to the reference and a value of 1 (red) is high genetic dissimilarity.

The multilocus phylogenetic tree exhibited a high degree of homoplasy with low bootstrap values (<70%) for many internal branches (Figure 3). Although unsupported by bootstrap analysis, two distinct clades were apparent. A large clade with short branch lengths comprising seven MLHs (H1, H92, H95, H96, H98, H106, and H111) included the Afla-Guard strain (H96) and other isolates predominantly in lineage IB (Figure 3). The other major clade with long and short branches included isolates that belonged to IB and IC lineages where the long branches are indicative of inter-lineage recombination; the AF36 biocontrol strain (H82) was in this clade. Patristic distances from the Afla-Guard reference isolate showed extensive clonality within IB (patristic distances close to 0 across the three loci) and recombination between IB and IC (incongruent patristic distances across the three loci; Figure 3). In *trpC*, both Afla-Guard and AF36 had a patristic distance of 0 which points to identical sequences at this locus; *mfs* showed the greatest sequence divergence from Afla-Guard for some isolates with patristic distances close to 1.

### Aflatoxin Contamination in Harvested Grain

Aflatoxin levels varied widely between states and were very low throughout the study. The only exception was in North Carolina in 2012, where the highest level of contamination was 103.8 ppb in the untreated plot (Table 8). Contamination levels in the remaining growing season-by-location combinations were very low at <12 ppb except in the untreated plots in Alabama in 2012. Significant differences (*P* < 0.05) in contamination between treated and untreated plots were observed only in North Carolina in 2012, while differences in the remaining growing season-by-location combinations were non-significant. Further, levels of aflatoxin contamination were lower in plots treated with Afla-Guard compared to plots treated with AF36, although these differences were not significant. For example, aflatoxin contamination was 2.75 and 4.75 ppb in plots treated with Afla-Guard and AF36, respectively, in North Carolina in 2012. A similar trend was also observed in 2013 in North Carolina, where aflatoxin contamination was 1.25 and 5.08 ppb in plots treated with Afla-Guard and AF36. Levels of aflatoxin contamination in Alabama in 2013 were below the minimum detection limit (Table 8).

**Table 8:** Aflatoxin concentration in harvested grain and dominant multilocus haplotypes (MLHs) of *Aspergillus flavus* in soil from fields in the southeastern United States treated with Afla-Guard and AF36 biocontrol strains.

Year	State	Treatment	Aflatoxin concentration (ppb)^x^		Dominant MLH^z^
			Afla-Guard^y^	AF36^y^	
2012	Alabama	Treated	5.96a	–	H96
		Untreated	27.85a	–	
	Georgia	Treated	–	–	H96
		Untreated	–	–	
	North Carolina	Treated	2.75a	4.75a	H96
		Untreated	103.75b	103.75b	
2013	Alabama	Treated	2.20a	1.28a	H96
		Untreated	2.04a	2.04a	
	Georgia	Treated	5.00a	–	H96
		Untreated	9.00a	–	
	North Carolina	Treated	1.25a	5.08a	H96
		Untreated	11.43a	11.43a	

x *Aflatoxin concentrations followed by the same letter are not significantly different at α = 0.05*.

y *Afla-Guard was not evaluated in Georgia in 2012, while AF36 was not evaluated in Alabama in 2012 and in Georgia in 2012 and 2013*.

z *H96 is the Afla-Guard MLH and belongs to lineage IB*.

## Discussion

Biocontrol using non-aflatoxigenic strains of *A. flavus* is considered the most successful option currently available to mitigate aflatoxin contamination of agricultural produce (Bhatnagar-Mathur et al., 2015; Ehrlich et al., 2015). Strains of *A. flavus* within a population vary in their ability to produce aflatoxins, ranging from individuals that do not produce the toxin (non-aflatoxigenic strains), to those that are potent producers of aflatoxins (Horn and Dorner, 1999). The non-aflatoxigenic chemotype is fairly common for the L-strain morphotype of *A. flavus* and the inability to produce the aflatoxins is the result of various deletions in the aflatoxin gene cluster (Chang et al., 2009). Application of non-aflatoxigenic strains that are capable of competitively excluding aflatoxigenic strains has been shown to be effective in reducing aflatoxin accumulation in maize in the United States (Dorner, 2009; Abbas et al., 2011b), Africa (Bandyopadhyay et al., 2016; Ayalew et al., 2017), and Europe (Mauro et al., 2018). However, neither of the non-aflatoxigenic strains in commercially available biocontrol products such as Afla-Guard or AF36, persist in soil and require annual applications to maintain their efficacy. As such, there has been considerable interest to understand factors that influence the efficacy of biocontrol treatments in an effort to develop biocontrol strategies that reduce aflatoxin accumulation at a greater rate but still persist in multiple years and generations of *A. flavus*. Haplotype diversity, mating type frequency and shifts in the populations of *A. flavus* were examined to assess the impact of applying biocontrol products, Afla-Guard and AF36, on the genetic structure of indigenous populations of *A. flavus* in maize fields in the southeastern United States.

*Aspergillus flavus* was the most frequently recovered species within *Aspergillus* section *Flavi* across all states before and after application of Afla-Guard and AF36, with all *A. flavus* isolates belonging to the L-strain morphotype. *A. parasiticus* was the second most recovered species with *A. caelatus, A. nomius*, and *A. tamarii* being recovered in very low frequencies only in Alabama. The high frequency of *A. flavus* relative to *A. parasiticus* or other species within section *Flavi* also has been reported in the southern United States (Horn and Dorner, 1998) and in Texas (Jaime-Garcia and Cotty, 2004), South America (Nesci and Etcheverry, 2002), and Africa (Hell et al., 2003; Atehnkeng et al., 2008). The predominance of *A. flavus* is due to its greater competitiveness and ability to survive better on crop debris than *A. parasiticus* or other species within *Aspergillus* section *Flavi* (Zummo and Scott, 1990). Warmer ambient air temperatures during this study were also more conducive for *A. flavus* that grows optimally at 37°C than for *A. parasiticus* that grows optimally at 25°C (Horn, 2005). This ecological niche adaptation explains why *A. parasiticus* is frequently associated with peanut pods in soil compared to above-ground crops such as maize and cotton. The high diversity in Alabama is consistent with reports of increased diversity within *Aspergillus* section *Flavi* in fields near 90° longitude in the southeastern United States and this diversity has been attributed to a combination of crop histories and crop response to environmental factors (Horn and Dorner, 1998). Generally, the frequency of *A. flavus* increased, while that of *A. parasiticus* decreased following application of biocontrol treatments. The increase in the densities of *A. flavus* may be due to other ecological factors rather than a simple dose-response to the introduction of biocontrol strains since 56–60% of individuals recovered after the biocontrol treatments were neither of the Afla-Guard nor the AF36 MLH.

Factors underlying shifts in the MLH diversity observed in this study are not known but could be related to sexual recombination within populations. *A. flavus* L is heterothallic with each individual strain having a single *MAT1-1* or *MAT1-2* mating type gene (Ramirez-Prado et al., 2008). In this study, *A. flavus* L populations exhibited a mating distribution consistent with ongoing sexual reproduction in as little as 2 weeks after biocontrol application. The only exceptions were two populations of *A. flavus* L in Alabama prior to biocontrol application in which individuals were significantly skewed toward *MAT1-1* in 2012 and *MAT1-2* in 2013. However, the mating-type distribution in these two populations in Alabama reverted to a 1:1 distribution of *MAT1-1:MAT1-2* at harvest. Thus, populations of *A. flavus* L in the southeastern United States are mainly sexual in nature as postulated earlier in a study that examined *A. flavus* populations from a peanut field in Georgia (Ramirez-Prado et al., 2008). Further evidence of sexuality in populations is indicated by the lack of a geographic structure between Alabama, Georgia, and North Carolina, which suggests gene flow and a largely panmictic population of *A. flavus* L. In addition, several strains with the genetic background of the Afla-Guard strain had either one of the two mating-types suggesting that either the Afla-Guard strain is recombining with the indigenous population of *A. flavus* or that the indigenous population is primarily of the IB lineage and is outcrossing. The proliferation and persistence of lineage IB isolates in soil suggests that it is possible to shift soil populations to the more non-aflatoxigenic IB lineage.

Sexual reproduction increases the diversity of aflatoxin profiles creating new vegetative compatible groups and sexuality is also associated with higher recombination rates in the aflatoxin cluster and less pronounced chemotype differences within the populations (Moore et al., 2009). Aflatoxin production in our sampled strains was not determined but an approximate *MAT1-1:MAT1-2* ratio of 1 in each state reported here suggests that populations of *A. flavus* L in the southeastern United States would exhibit variability in aflatoxin concentrations. The potential of a biocontrol strain to recombine with predominantly aflatoxigenic native strains is greater when the *A. flavus* population has equal distribution of *MAT1-1* and *MAT1-2* (Moore et al., 2013) and this has direct implications in selection of non-aflatoxigenic strains. Sexual crosses result in a higher frequency of aflatoxigenic progeny strains when the AF36 strain is the parental strain and a lower frequency of aflatoxin producing progeny strains when the Afla-Guard strain is the parent (Olarte et al., 2012). Unlike the Afla-Guard strain, the AF36 strain has a full aflatoxin gene cluster and replacement with a functional *pskA* can promote synthesis of aflatoxin in AF36 progeny strains. Thus, non-aflatoxigenic strains that lack the cluster gene such as the Afla-Guard strain and similar members within lineage IB, that are likely to recombine with predominant aflatoxigenic strains will be preferable in enhancing the efficacy and sustainability of biocontrol of aflatoxin accumulation.

While clone corrected populations showed a near 1:1 distribution of the two mating types, the frequency of uncorrected *MAT1-1* and *MAT1-2* individuals was significantly skewed toward *MAT1-2* in Alabama and North Carolina and toward *MAT1-1* in Georgia. This skewed distribution to one mating-type can partly be explained by clonal reproduction of a specific vegetative compatibility group that has an advantage over others during vegetative propagation (Leslie and Klein, 1996). The enrichment of either *MAT1-1* or *MAT1-2* in the population also may be due to differences in female fertility or fitness associated with either mating-type (Leslie and Klein, 1996; Moore et al., 2013). Dominance of a specific mating-type suggests that *A. flavus* L populations can be predominantly clonal despite the presence of sexual reproduction, as reported in the pathogenic fungus *Penicillium marneffei* (Henk et al., 2012). The skew toward either *MAT1-1* or *MAT1-2* though not significant after clone correction, can inform selection of non-aflatoxigenic strains in the design of sustainable biocontrol strategies to mitigate aflatoxin accumulation. For example, if a population is predominantly *MAT1-1* as observed in the clonal population of *A. flavus* in Argentina (Moore et al., 2013), then a *MAT1-2* biocontrol strain would be better because there would be more opportunities for sex. While a high frequency of female sterility can ultimately drive a sexually recombining population to clonality (Hornok et al., 2007), the frequency of *MAT1-1* or *MAT1-2* individuals in field populations examined in the present study was approximately equal after clone correction. This suggests that female fertility in *A. flavus* populations was sufficiently high to achieve mating type equilibrium across all three states. Sex can contribute to making biocontrol more sustainable by spreading determinants of non-aflatoxigenicity to subsequent *A. flavus* generations.

Genotyping *A. flavus* field populations before and after biocontrol treatments provides valuable information on the availability and fitness of the biocontrol strain during the growing season and its impact on changing the composition of indigenous populations of *A. flavus* in the soil. Frequently recovered biocontrol strains are likely to persist in soil and be more effective in reducing aflatoxin accumulation over several generations of *A. flavus*. In this study, most of the *A. flavus* L strains recovered after application of treatments belonged to the same MLH as Afla-Guard strain, while very few strains belonged to the same MLH as the AF36 strain. The Afla-Guard haplotype H96 belongs to the IB lineage, while the AF36 MLH H82 belongs to the IC lineage (Geiser et al., 2000). Our data also indicated that both intra- and inter-lineage recombination generates extensive diversity in *A. flavus* with many MLHs sampled only once. This is not surprising given that soil population densities increased several fold over the course of the season. These results are consistent with a recent study that identified two distinct *A. flavus* populations that were widespread in the United States, where one of the populations was highly clonal and another was more diverse (Drott et al., 2019). While the use of microsatellite markers precluded conclusive evidence of recombination and genetic lineage structuring (Drott et al., 2019) it is clear from the present study that *A. flavus* L populations are structured by lineage (IB and IC) and undergoing intra- and inter-lineage recombination. For example, the results from patristic analysis showed that Afla-Guard (a member of IB) and AF36 (a member of IC) are identical for sequence variation in *trpC*, which was reported previously (Moore et al., 2009). This is expected with ongoing genetic exchange and recombination in field populations and indicates the need to examine more genetic markers to fully determine levels of admixture in populations. Specifically, studies examining single nucleotide polymorphisms from more loci and genome-wide (Geiser et al., 1998, 2000; Taylor et al., 1999; Moore et al., 2009, 2013, 2017; Okoth et al., 2018) are necessary for ultimately tracking the fate of released *A. flavus* biocontrol strains and their potential to shift the relative frequencies of IB and IC lineages.

The complete MLH data from North Carolina allows us to examine the competitiveness and survival of *A. flavus* L individuals in lineages IB and IC between study years. At the end of 2012, 62% of isolates were identical to the Afla-Guard MLH, while only 2% were identical to the AF36 MLH. Prior to biocontrol treatments in the 2013, 15% of the isolates were identical to Afla-Guard haplotype but none were identical to the AF36 haplotype. This suggests that *A. flavus* L individuals in the IB lineage may be more competitive and survive better than those in the IC lineage in the geographical region sampled. These findings indicate that Afla-Guard is more effective than AF36 in shifting the indigenous soil population of *A. flavus* toward the IB lineage. The ability of the Afla-Guard strain to shift soil populations toward the IB lineage could be because the strain is more viable and sexually fertile than the AF36 strain such that both asexual and sexual reproduction results in individuals with a MLH that is similar to that of Afla-Guard. The lower fertility or viability of the AF36 strain seems to be supported by the observation that only 2 of the 16 strains with the AF36 MLH H82 were *MAT1-1*.

Non-aflatoxigenic strains of *A. flavus* in the IB lineage with a MLH similar to that of Afla-Guard strain are expected to be more effective than those in the IC lineage with the AF36 MLH in reducing aflatoxin accumulation in the southeastern United States. Non-aflatoxigenic strains within lineage IB may further be maintained by balancing selection acting to maintain the non-aflatoxigenic phenotype in *A. flavus* populations (Moore et al., 2009; Drott et al., 2017). Thus, use of non-aflatoxigenic strains in lineage IB such as the Afla-Guard strain is expected to be more effective in reducing aflatoxin accumulation over several generations of *A. flavus*. Our prediction of Afla-Guard to be more effective than AF36 in the southeastern United States is supported by previous studies in the region (Abbas et al., 2011a,b; Meyers et al., 2015). Given that the Afla-Guard strain was isolated in Georgia, it is also highly possible that the strain is well-adapted in the region compared to the AF36 strain, which would also partly explain why the AF36 MLH was either recovered in very low frequency or not recovered at harvest. Use of locally or regionally adapted non-aflatoxigenic strains is also desirable as it would favor sexual recombination with indigenous aflatoxigenic strains and result in more a sustainable biocontrol strategy.

The low levels of aflatoxin contamination observed in this study do not allow for a direct assessment of the impact of the shifts in the genetic structure of *A. flavus* on the levels of aflatoxin in maize. In maize, aflatoxin contamination is often associated with heat and drought stress (Windham et al., 2009) especially during reproductive growth with temperatures of 37°C being optimum for the fungus. Here, variations in temperature and rainfall appeared to correlate with levels of aflatoxin. The highest level of contamination in 2012 in North Carolina was primarily due to the high temperature during the reproductive period of maize. Similarly, little to no contamination was observed in 2013 due to the high precipitation and comparatively lower temperatures. Field trials involving large-scale plots where biocontrol treatments are separated by larger buffer zones under conditions that favor aflatoxin accumulation over several seasons will be needed to better assess this impact. In addition, aflatoxin production will need to be determined for sampled strains and lineages to fully understand the relationship between *A. flavus* aflatoxin producing potential and population genetic structure. Ultimately, population genetic data will need to be combined with data on the ecological adaptation of the selected non-aflatoxigenic strains from different environments and crop production systems. While increasing the efficacy of biocontrol of aflatoxin accumulation in maize is important, it is apparent that the population biology of *A. flavus* in the soil will play a critical role in the design of more sustainable biocontrol strategies.

## Data Availability

The datasets generated for this study can be found in the Sequences used for MLST (AF17, *mfs*, and *trpC*) were submitted to GenBank under accession numbers 2232583, 2233208, and 223307.

## Author Contributions

IC and PO conceived the experiments. IC, PO, and GP designed the experiments. ML performed the experiments, collected, and analyzed data. KB, AH, RK, and RH performed experiments and collected data. JL assisted with data analysis. ML, IC, JL, GP, KB, AH, RK, RH, and PO contributed to writing and approved the final manuscript.

### Conflict of Interest Statement

The authors declare that the research was conducted in the absence of any commercial or financial relationships that could be construed as a potential conflict of interest.

## Supplementary Material

Table S1 Identity metadata of isolates of *Aspergillus flavus* used to study shifts in *A. flavus* soil populations following application of biocontrol strains.
